# Increased sensitivity to gemcitabine of P-glycoprotein and multidrug resistance-associated protein-overexpressing human cancer cell lines

**DOI:** 10.1038/sj.bjc.6601011

**Published:** 2003-06-10

**Authors:** A M Bergman, H M Pinedo, I Talianidis, G Veerman, W J P Loves, C L van der Wilt, G J Peters

**Affiliations:** 1 Department Medical Oncology, VU University Medical Center, PO Box 7057, 1007 MB Amsterdam, The Netherlands; 2 Institute of Molecular Biology and Biotechnology, FORTH, 1527 Vassilika Vouton, 71110 Herakleion Crete, Greece

**Keywords:** gemcitabine, multidrug resistance, collateral sensitivity, deoxycytidine kinase, thymidine kinase

## Abstract

Gemcitabine (2′,2′-difluorodeoxycytidine) is a deoxycytidine analogue that is activated by deoxycytidine kinase (dCK) to its monophosphate and subsequently to its triphosphate dFdCTP, which is incorporated into both RNA and DNA, leading to DNA damage. Multidrug resistance (MDR) is characterised by an overexpression of the membrane efflux pumps P-glycoprotein (P-gP) or multidrug resistance-associated protein (MRP). Gemcitabine was tested against human melanoma, non-small-cell lung cancer, small-cell lung cancer, epidermoid carcinoma and ovarian cancer cells with an MDR phenotype as a result of selection by drug exposure or by transfection with the *mdr1* gene. These cell lines were nine- to 72-fold more sensitive to gemcitabine than their parental cell lines. The doxorubicin-resistant cells 2R120 (MRP1) and 2R160 (P-gP) were nine- and 28-fold more sensitive to gemcitabine than their parental SW1573 cells, respectively (*P*<0.01), which was completely reverted by 25 *μ*M verapamil. In 2R120 and 2R160 cells, dCK activities were seven- and four-fold higher than in SW1573, respectively, which was associated with an increased dCK mRNA and dCK protein. Inactivation by deoxycytidine deaminase was 2.9- and 2.2-fold decreased in 2R120 and 2R160, respectively. dFdCTP accumulation was similar in SW1573 and its MDR variants after 24 h exposure to 0.1 *μ*M gemcitabine, but dFdCTP was retained longer in 2R120 (*P*<0.001) and 2R160 (*P*<0.003) cells. 2R120 and 2R160 cells also incorporated four- and six-fold more [^3^H]gemcitabine into DNA (*P*<0.05), respectively. P-glycoprotein and MRP1 overexpression possibly caused a cellular stress resulting in increased gemcitabine metabolism and sensitivity, while reversal of collateral gemcitabine sensitivity by verapamil also suggests a direct relation between the presence of membrane efflux pumps and gemcitabine sensitivity.

Gemcitabine (2′,2′-difluorodeoxycytidine) is a deoxycytidine analogue with proven activity in ovarian, pancreatic and non-small cell lung cancer (NSCLC) *in vivo*, *in vitro* and in the clinic (Abratt *et al*, 1994; Lund *et al*, 1994; Braakhuis *et al*, 1995; Heinemann, 2001). Deoxycytidine kinase (dCK) phosphorylates both deoxycytidine (dCyd) and gemcitabine to their monophosphates, which are subsequently phosphorylated to deoxycytidine triphosphate (dCTP) and gemcitabine triphosphate (dFdCTP), respectively. Gemcitabine can be inactivated by deamination catalysed by deoxycytidine deaminase (dCDA) (Heinemann *et al*, 1988). dFdCTP can be incorporated into both DNA and RNA (Huang *et al*, 1991; Ruiz van Haperen *et al*, 1993b). The mitochondrial enzyme thymidine kinase 2 (TK2) phosphorylates the natural nucleosides thymidine and deoxycytidine, but also gemcitabine, although, to a lesser extent than dCK (Eriksson *et al*, 1991; Bergman *et al*, 1999). This is in contrast to the cytosolic enzyme thymidine kinase 1, which does not phosphorylate deoxycytidine (Eriksson *et al*, 1991). Since dCTP is the major natural feedback inhibitor of dCK and competes with dFdCTP for DNA polymerase, an increase in dCTP pools will decrease gemcitabine sensitivity (Heinemann *et al*, 1990; Ruiz van Haperen and Peters, 1994).

Crossresistance to some structurally and functionally unrelated natural-derived drugs (e.g. daunomycin, etoposide, vincristine) is called multidrug resistance (MDR). Multidrug resistance is caused by overexpression of the plasma membrane drug efflux pumps P-glycoprotein (P-gP), the product of the *mdr1* gene, and multidrug resistance-associated protein (MRP) (Endicott and Ling, 1989; Grant *et al*, 1994). These pumps can be blocked by verapamil (Cornwell *et al*, 1987). The MRP family currently has seven members, all with different drug specificities (Borst *et al*, 2000). Although gemcitabine is a substrate for MRP5 efflux pump (Davidson *et al*, 2002), it is predominantly transported into the cell across cell membranes via facilitated diffusion (equilibrative nucleoside transporter, ENT) and sodium-dependent concentrative mechanisms (concentrative nucleoside transporter, CNT) (Mackey *et al*, 1998, 1999; Ritzel *et al*, 2001). Jensen *et al* (1997) observed that some small-cell lung cancer (SCLC) cells with P-gP overexpression were more sensitive to gemcitabine and the structurally related deoxycytidine analogue 1-*β*-D-arabinofuranosylcytidine (cytarabine, ara-C), although Grant *et al* (1995) observed crossresistance to ara-C in a P-gP-overexpressing leukaemia cell line.

We tested the sensitivities to gemcitabine of various pairs of cell lines and their MDR variants derived from different human tumours. The human NSCLC cell line SW1573 and its P-gP- and MRP1-overexpressing variants were used for mechanistic studies on gemcitabine sensitivity of MDR cells. Moreover, a possible role of the membrane efflux pumps in gemcitabine sensitivity was investigated.

## MATERIALS AND METHODS

### Chemicals and reagents

Dulbecco's modified eagle's medium (DMEM) and Rosswell Park Memorial Institute (RPMI) medium were purchased from Flow Laboratories (Irvine, UK); foetal calf serum from Life Technologies (New York, NY, USA); trichloroacetic acid (TCA), glutamine and gentamicin from Merck (Darmstadt, Germany); trypsin and sulphorhodamine B (SRB) from Sigma Chemical Co. (St Louis, USA); and VP-16 (etoposide) from Bristol-Myers Squibb (Weesp, the Netherlands). Eli Lilly (Indianapolis, IN, USA) kindly supplied gemcitabine and [5-^3^H]gemcitabine (16.7 Ci mmol^−1^). [5-^3^H]Deoxycytidine was purchased from Moravek (Brea, CA, USA) [2-^14^C]thymidine (58.8 Ci mmol^−1^) was from Dupont de Nemours NEND (Dreiech, Germany) and [5-^3^H]uridine (27.8 Ci mmol^−1^) from Amersham International (Buckinghamshire, England). All other chemicals were of analytical grade and commercially available.

### Cell culture

The *in vitro* experiments were performed with five parental cell lines and eight MDR variants (Table 1

**Table 1:** MDR phenotype, sensitivity to gemcitabine and sensitivity factors of human cancer cell lines

**Cell line**	**Origin**	**MDR phenotype**	**Doubling time (h)**	**IC_50_ (nM) gemcitabine**	**Sensitivity factor**
SW1573	NSCLC	Parental	35±5	17.0±2	1
2R120^a^		DOX resistant, MRP1	50±6	1.9±1.2	8.9***
2R160^a^		DOX resistant, P-gP	50±6	0.6±0.4	27.9***
SW1573/S1(1.1)^b^,^c^		P-gP transfected	30±2	5.7±2.4	3.0**
SW1573/S1(MRP)^b^, ^c^		MRP1 transfected	35±8	0.05±0.02	340***

GLC4	SCLC	Parental	22±5	4.1±0.6	1
GLC4/ADR^b^		DOX resistant, MRP1	31±9	0.4±0.2	11*

A2780	Ovarian	Parental	22±9	1.6±0.3	1
2780AD^d^		DOX resistant, P-gP	26±8	0.03±0.03	55**

KB3-1	Epidermoid	Parental	16±3	59±24	1
KB8-5^e^		Colchicine resistant, P-gP	19±5	33.0±20.	1.8 NS

BRO	Melanoma	Parental	21±3	300±87	1
BROmdr^f^		P-gP transfected	29±4	33±12	9.0**

MDR=multidrug resistance; NSCLC=non-small-cell lung cancer; SCLC=small-cell lung cancer; DOX=doxorubicin; P-gP=P-glycoprotein; MRP=multidrug resistance-associated protein. Cells were exposed to gemcitabine for 72 h. Values represent the mean IC_50_±s.d. of at least three experiments. IC_50_=50% growth-inhibiting concentration, sensitivity factor; IC_50_ parental/IC_50_ MDR variant. Statistical analysis by *t*-test (independent samples); NS=not significant.

* *P*<0.05.

** *P*<0.01.

*** *P*<0.001. References:

a Kuiper *et al* (1990).

b Zaman *et al* (1993).

c Zaman *et al* (1994).

d Louie *et al* (1986).

e Shen *et al* (1986).

f Lincke *et al* (1990).

). All cell lines were grown in monolayers in DMEM, except for GLC4S and GLC4/ADR, which grew as semimultilayers in RPMI, at 37°C and 5% CO_2_. These cells grow in several layers, but are only loosely attached to the flask. Both media were supplemented with 7.5% heat-inactivated foetal calf serum, and 250 ng ml^−1^ gentamicin. Cells were regularly screened for *Mycoplasma* contamination by using a rapid detection system with a ^3^H-labelled DNA probe (Gen-Probe, San Diego, CA, USA) and were found to be negative.

### Chemosensitivity testing

The determination of the IC_50_ (the drug concentration causing 50% growth inhibition) in monolayer cell lines was performed using the SRB assay. The assay was performed using the NCI protocol with some small modifications (Skehan *et al*, 1990; Keepers *et al*, 1991). For GLC4S and GLC4/ADR, both growing as semimultilayers, the tetrazolium (MTT) assay was performed as previously described (Keepers *et al*, 1991). Culture conditions were optimised for all cell lines. For both assays, the cells were plated in 96-well plates at day 1, in different densities, depending on their doubling times (4000–15 000 cells per well). The optimal plating number was the highest number of cells, which enabled log-linear growth for 4 days. Log-linear growth or exponential growth is the phase of growth in which each descendant of the parental cell will divide as well.

On day 2, cells were exposed to gemcitabine (final concentrations ranged from 5 × 10^−16^ to 5 × 10^−5^ M), with or without 25 *μ*M verapamil for 72 h and optical density (OD) was estimated either by the SRB or MTT assay and set at 100%; the OD of cells at the day of drug administration was set at 0%. The IC_50_ was the drug concentration resulting in a relative OD of 50%, total growth inhibition when OD was similar to the initial value, 0%, and an OD lower than the initial value 0% represents cell kill (Peters *et al*, 1993).

### dCK enzyme activity

For determination of dCK activities, 10 000 **g** supernatants were prepared with cold dCK buffer, containing 0.3 M Tris-HCl (pH 8.0), essentially as described (Ruiz van Haperen *et al*, 1993a). Protein content was estimated with the Biorad protein assay. To 25 *μ*l of supernatant containing 0.2–4.0 × 10^5^ cells, 25 *μ*l of a substrate mixture was added (final concentrations: 10 mM ATP, 5 mM MgCl_2_, 30 mM Tris-HCl and 230 *μ*M ^3^H-dCyd (final specific activity 0.04 Ci mmol^−1^)), with or without 1 mM thymidine to inhibit TK2-mediated phosphorylation of dCyd (Eriksson *et al*, 1991), and incubated at 37°C for 15 min. The radio-labelled product dCMP was quantitated in a liquid scintillation counter, after thin layer chromatography on polyethyleneimine cellulose layers.

### dCDA enzyme activity

Activity of dCDA was determined as described earlier (Ruiz van Haperen *et al*, 1993a). Briefly, 10 000 **g** supernatants were prepared and enzyme assays were performed at 37°C in 1–5 × 10^6^ cells with 500 *μ*M dCyd as a substrate for 15 or 25 min. The deaminated product deoxyuridine was analysed using reversed phase high-performance liquid chromatography (HPLC) (Ruiz van Haperen *et al*, 1993a).

### Quantitative reverse transcriptase–polymerase chain reaction assay for dCK-mRNA with competitive templates

RNA was extracted from confluent growing cells using the RNAzole method and cDNA samples were prepared as previously described (Rots *et al*, 2000). Competitive template reverse transcriptase–polymerase chain reaction (RT–PCR) assays for dCK mRNA and the housekeeping gene *β*-actin were performed as described previously (Kroep *et al*, 2002). Levels of expression were reported as units of dCK-mRNA/10^6^ *β*-actin mRNA molecules.

### Western blot for dCK

Western blotting by affinity-purified rabbit antibody against human dCK was performed as previously described (Hatzis *et al*, 1998).

### dFdCTP accumulation and retention

For dFdCTP accumulation and retention, cells were plated in six-well plates at 0.5–1.0 × 10^6^ cells per well in 2 ml medium, and exposed to 0.1 or 1.0 *μ*M gemcitabine for 24 h. For the retention, cells were cultured in drug-free medium for 4 or 24 h after incubation. After harvesting, nucleotides were extracted and quantitated with HPLC as previously described (Ruiz van Haperen *et al*, 1994).

### [^3^H]Gemcitabine incorporation into DNA and RNA

Incorporation studies were performed using 96-well filter plates essentially as described previously (Van der Wilt *et al*, 1993; Van Moorsel *et al*, 1999a). A total of 1.0 × 10^5^ cells per well were plated in a volume of 100 *μ*l, whereafter 100 *μ*l of [^3^H]gemcitabine containing medium (4 Ci mmol^−1^) was added resulting in final concentrations of 0.1 or 1.0 *μ*M. After 24 h incubation, the medium was removed by suction through the filters. Filters were washed with TCA and phosphate-buffered saline (PBS), followed by incubation with RNAse A or without RNAse A at 37°C. After termination of the reaction, the filters were washed again with TCA, H_2_O and 70% ethanol, removed and radioactivity was counted as described previously (Van Moorsel *et al*, 1999a). Counts in wells incubated with RNAse A were considered to represent [^3^H]gemcitabine incorporated into DNA, the difference in counts between incubation with or without RNAse A was considered to represent [^3^H]gemcitabine incorporation into RNA. To correct for DNA and RNA synthesis, experiments were performed simultaneously with cells exposed to 0.1 or 1.0 *μ*M cold gemcitabine and 5.6 *μ*M [^14^C]thymidine (62.8 mCi mmol^−1^) or 0.165 *μ*M [^3^H]uridine (25 Ci mmol^−1^). Cell numbers were estimated by performing this experiment with cells exposed to unlabelled gemcitabine in a concentration of 0.1 or 1.0 *μ*M (Van Moorsel *et al*, 1999a).

### Fluorometric analysis of DNA unwinding assay

To measure the gemcitabine-induced DNA damage, the fluorometric analysis of DNA unwinding (FADU) assay was used (Birnboim and Jevcak, 1981; Bergman *et al*, 1996). Essentially, 3 × 10^6^ cells were incubated for 24 h at 37°C with gemcitabine in a concentration of 0.1 or 1.0 *μ*M. Cells exposed to 50 *μ*M VP-16 for 24 h were used as a positive control, since this drug is known to introduce double-strand (ds) DNA breaks very effectively, and cells not exposed to drugs were used as a negative control. Cells were lysed and exposed to an alkaline environment, allowing the DNA to unwind. The extent of strand breaks in the DNA determines the extent of DNA unwinding at the end of incubation. The dsDNA was stained by ethidium bromide and the ratio of fluorescence between treated and control samples was used to determine the percentage dsDNA (Bergman *et al*, 1996).

### Statistical analysis

In case of significant differences between parental and MDR cells, a *t*-test was used to compare unpaired data of the two cell lines. The computer program SPSS (version 7.5, SPSS, Inc., Chicago, IL, USA) was used for statistical analysis.

## RESULTS

### Growth inhibition tests

**Figure 1:**
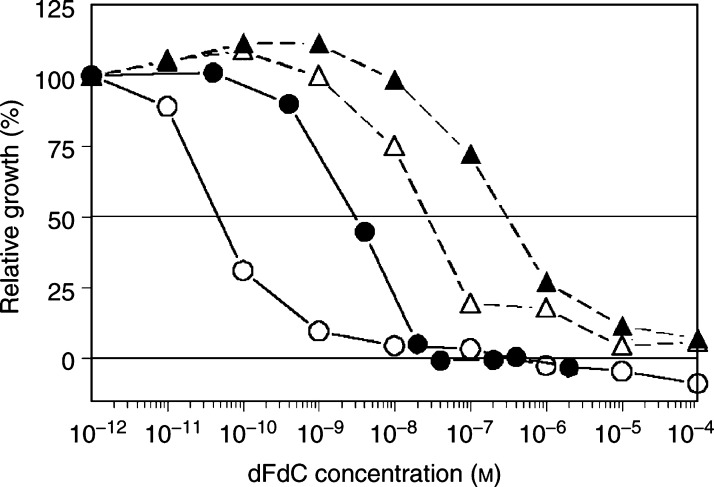
Representative growth inhibition curves of the human melanoma cell line BRO (-▴-) and its transfected variant BROmdr (-▵-) (broken line) and the human ovarian carcinoma cell line A2780 (-•-) and its doxorubicin-resistant, P-gP-overexpressing variant A2780AD (-○-) (solid line). Cells were exposed to gemcitabine for 72 h.

The parental cell lines showed large differences in sensitivity to gemcitabine, which was not related to their doubling time, this indicates that differences in metabolic activation and mechanisms of action are predominantly responsible for sensitivity to gemcitabine. As a control, clonogenic assays were performed for some cell lines as described previously (Bergman *et al*, 2000), which also showed a similar difference in sensitivity (data not shown). SW1573 cells were intermediately sensitive to gemcitabine compared to the other parental cells; BRO, GLC4, KB3-1 and A2780 cells. All SW1573 variants were significantly (*P*<0.01) more sensitive to gemcitabine than the parent (Figure 1

, Table 1). 2R120 and 2R160 cells, the MRP1- and P-gP-overexpressing variants of SW1573, respectively, were nine- and 28-fold more sensitive to gemcitabine, respectively. The P-gP-transfected variant S1(1.1) was three-fold more sensitive, but the largest difference was found for the MRP1-transfected S1(MRP) cells, which were 340-fold more sensitive to gemcitabine than SW1573 cells (*P*<0.001). The doxorubicin-selected, MRP1-overexpressing variant of GLC4, GLC4/ADR, was 11.1-fold more sensitive to gemcitabine than its parental cells (*P*<0.05). The human ovarian cancer A2780 cells were the most sensitive parental cells, but the doxorubicin-resistant P-gP-overexpressing variant 2780AD was even more sensitive to gemcitabine (*P*<0.01). KB8-5 cells were included since P-gP was induced by exposure to colchicine; also this phenotype was associated with increased sensitivity to gemcitabine. BRO cells were the least sensitive of the parental cells tested, but its mdr1-transfected variant was significantly more sensitive (*P*<0.01).

**Figure 2:**
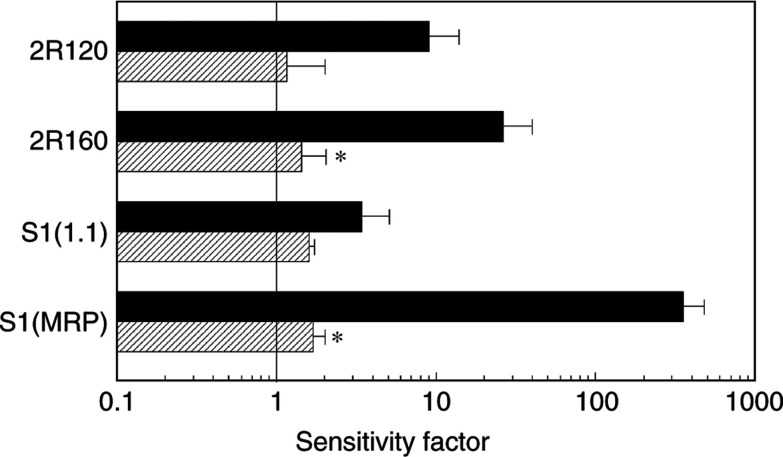
Sensitivity factors to gemcitabine, without 25 *μ*M verapamil (▪) or with 25 *μ*M verapamil (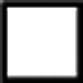) (IC_50_ of the MDR variants relative to IC_50_ of SW1573 was set at 1), of the MRP1-overexpressing 2R120, the P-gP-overexpressing 2R160, the mdr1-transfected S1(1.1) and the MRP1-transfected S1(MRP). Verapamil itself did not affect cellular growth. Values are means±s.d. of at least three experiments. ^*^Sensitivity with 25 *μ*M verapamil statistically significantly different from gemcitabine alone (*t*-test, independent samples, *P*<0.05).

The MDR phenotype of doxorubicin resistance can be reversed by the calcium-channel blocker verapamil. This phenomenon has been used as a characteristic of the efflux pump. In order to determine whether the activity of the pump was associated with the collateral sensitivity to gemcitabine, we exposed SW1573 and its variants to gemcitabine and verapamil. Verapamil almost completely reversed the gemcitabine sensitivity to the level of the parental SW1573 cells (Figure 2

). This reversal was significant in 2R160 and S1(MRP) cells (*P*<0.05).

### Deoxycytidine kinase, thymidine kinase 2 and deoxycytidine deaminase assays

Since gemcitabine cytotoxicity is dependent on its phosphorylation, we determined the activities of the enzymes involved in this process. SW1573 cells had the lowest dCK activity, this activity was 6.6- and 4.0-fold higher in 2R120 (*P*<0.001) and 2R160 cells (not significant), respectively (Table 2

**Table 2:** dCK, TK2 and dCDA activities and dCK protein and mRNA expression in the human NSCLC cell line SW1573 and its doxorubicin MDR variants; 2R120 (MRP1) and 2R160 (P-gP)

**Enzyme**		**SW1573**	**2R120**	**2R160**
dCK	Activity	0.3±0.08	2.1±0.3***	1.3±0.7 NS
	Protein	0.3	2.0	0.6
	mRNA	0.5	3.1	2.8

TK2		0.2±0.2	0.2±0.3	0.1±0.2 NS

dCDA		37±3.0	13±3***	17±1***

Enzyme activities are means±s.d. of at least three experiments in nmol h^−1^ per 10^6^ cells. For dCK and TK2 activity, dCyd was used as a substrate with or without thymidine to block TK2 activity. The difference in dCMP formation between dCyd as a substrate and dCyd and thymidine as a substrate was considered as TK2 activity. The protein content of SW1573, 2R120 and 2R160 cells was about 273±77, 442±152 and 210±8 *μ*g per 10^6^ cells, respectively (means±s.d.). Protein and mRNA expression are means of two and three separate experiments in (OD mm^−2^) and (dCK/*β*-actin × 10^−3^), respectively. Statistically different from SW1573 by *t*-test (independent samples); NS=not significant.

*** *P*<0.001.

). In 2R120 cells, TK2 activity was unchanged compared to its parental SW1573 cells. However, TK2 activity was two-fold lower in 2R160 than in SW1573 cells (not significant).

The amount of dCK protein present in the cell as determined with Western blots, revealed a 7.2- and 2.2-fold higher expression in 2R120 and 2R160 cells, respectively, than in the parental SW1573 cells (Table 2). In order to determine whether the increase in dCK protein was the result of increased expression, we determined the expression of dCK-mRNA (Table 2). The dCK/*β*-actin ratio was 6.1- and 5.5-fold higher in 2R120 and 2R160 cells than in the parental SW1573 cells, respectively (*P*<0.001).

dCDA activity was decreased 2.9- and 2.2-fold in 2R120 and 2R160 cells, respectively (Table 2).

### dFdCTP accumulation and retention

**Figure 3:**
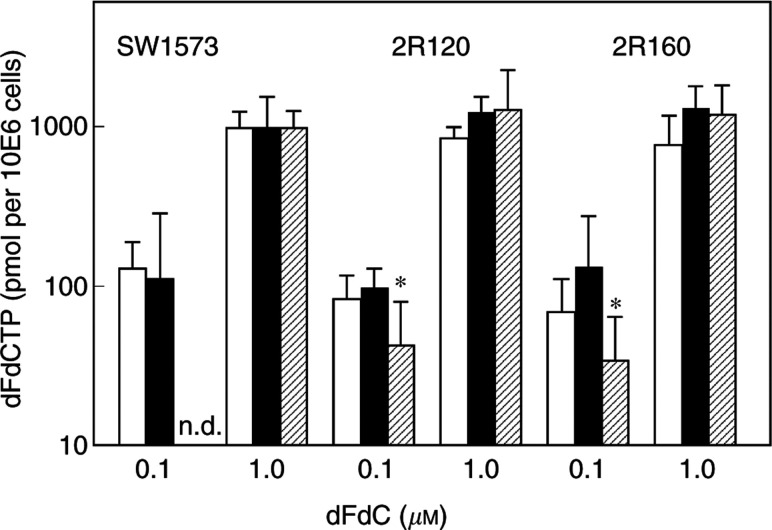
Accumulation (□), of dFdCTP after 24 h exposure to 0.1 or 1.0 *μ*M gemcitabine and its retention after suspension in drug-free medium for 4 h (▪) or 24 h (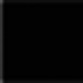) in the human NSCLC cell line SW1573 and its doxorubicin-resistant MDR cell lines; MRP1-overexpressing 2R120 and P-gP-overexpressing 2R160. Values are means±s.d. of at least three experiments. ^*^Statistically significant different from SW1573 cells (*t*-test, independent samples, *P*<0.005). n.d.=not detectable.

The active metabolite of gemcitabine is dFdCTP. Besides its accumulation, retention is also important for the cytotoxicity of gemcitabine. No significant difference in dFdCTP accumulation was found between the cell lines, both at 0.1 and 1.0 *μ*M gemcitabine (Figure 3

). In all cells, accumulation was concentration dependent, six- to eight-fold more dFdCTP accumulated at 1.0 *μ*M than at 0.1 *μ*M gemcitabine. At 0.1 *μ*M gemcitabine, dFdCTP was retained longer in 2R120 (*P*=0.001) and 2R160 (*P*=0.003) cells than in the parental cell line SW1573. At 1.0 *μ*M gemcitabine, no difference in retention of dFdCTP was found between the cell lines. Another active metabolite of gemcitabine, gemcitabine-diphosphate was not detectable.

### Gemcitabine incorporation into DNA and RNA, and DNA damage

Following its accumulation, dFdCTP may be incorporated into DNA and RNA. The extent and retention are dependent on DNA and RNA polymerases, but also on subsequent excision. We not only determined the total extent of incorporation but also its relation to synthesis of DNA and RNA, which are inhibited by gemcitabine. SW1573, 2R120 and 2R160 cells not exposed to gemcitabine incorporated 53±29, 8.7±5.0 and 41±9 pmol thymidine h^−1^ per 10^6^ cells into DNA and 2.5±0.7, 2.7±2.9 and 3.1±0.5 pmol uridine h^−1^ per 10^6^ cells into RNA, respectively. At 0.1 *μ*M gemcitabine, inhibition of DNA synthesis varied between 79 and 91%, and at 1 *μ*M gemcitabine between 94 and 97%. For inhibition of RNA synthesis, these values were less pronounced and varied between 0 and 4.7%, and 4.6 and 14.5%, respectively, for the three cell lines.

**Figure 4:**
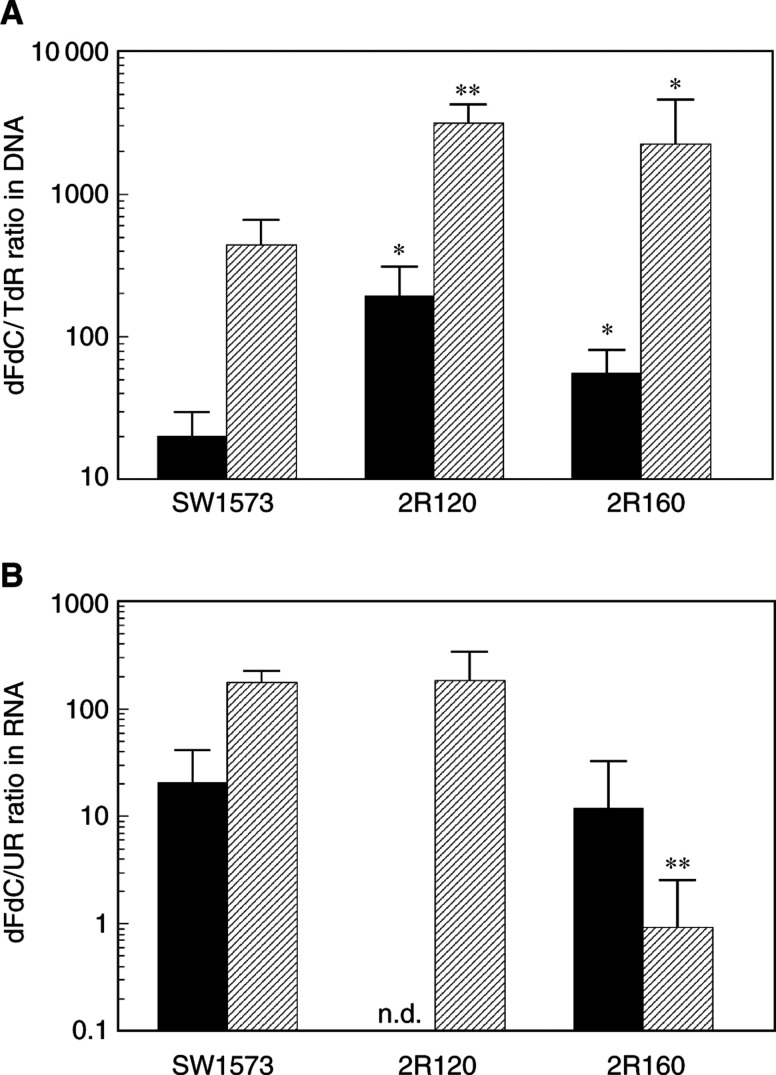
Incorporation of [^3^H]gemcitabine into DNA relative to incorporation of [^14^C]thymidine (TdR) into DNA (**A**) and incorporation of [^3^H]gemcitabine into RNA relative to incorporation of [^3^H]uridine (UR) into RNA (**B**) after 24 h exposure to 0.1 *μ*M (▪) or 1.0 *μ*M (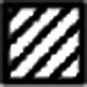) gemcitabine in the human NSCLC cell line SW1573 and its doxorubicin-resistant MDR cell lines; MRP1-overexpressing 2R120 and P-gP-overexpressing 2R160. Values are means±s.d. of at least three experiments. ^*^Statistically significant different from SW1573 cells (*t*-test, independent samples), *P*<0.05, ^**^P<0.02. n.d.=not detectable.

When corrected for the inhibition of DNA synthesis, [^3^H]gemcitabine incorporation into DNA was greater at the higher concentrations in all three cell lines, suggesting that incorporation was concentration dependent (Figure 4

). At 0.1 *μ*M gemcitabine 2R120 and 2R160 cells incorporated 9.6- (*P*=0.04) and 2.8-fold and at 1.0 *μ*M gemcitabine 7.1- (*P*=0.01) and 5.1-fold more [^3^H]gemcitabine into DNA than SW1573 cells, respectively. When corrected for inhibition of RNA synthesis 1 *μ*M [^3^H]gemcitabine incorporation into RNA was about 100-fold lower in 2R160 compared to SW1573 and 2R120 cells (data not shown).

Incorporation of gemcitabine may cause DNA damage, which is the result of the extent of its incorporation and efficacy of the DNA repair mechanisms. In SW1573 and 2R120 cells, the amount of dsDNA breaks was concentration dependent (7 and 9% dsDNA breaks at 0.1 *μ*M, 16 and 39% ds breaks at 1 *μ*M gemcitabine, respectively). However, in 2R160 comparable DNA damage was found at 0.1 and 1.0 *μ*M gemcitabine (19 and 25% dsDNA breaks, respectively).

## DISCUSSION

In this paper, we describe an increased collateral sensitivity to gemcitabine in five pairs of cell lines with an MDR phenotype. Collateral sensitivity is defined as an increased sensitivity to one class of drugs, while made resistant to another class of drugs. This increased sensitivity was associated with an increase in the activity of dCK in the variant cell lines. Apparently, the increased dCK activity was related to an upregulation of the gene, reflected by an increased mRNA and protein content of the cells. Moreover, the increased dCK activity was stable for many passages, indicating the structural nature of the upregulation of the gene. Deoxycytidine kinase plays a pivotal role in gemcitabine activation since it catalyses the first step in phosphorylation, which is rate limiting for further phosphorylation to active metabolites. This is based on the observation that dCK deficiency is associated with gemcitabine resistance (Bergman *et al*, 1999, 2000) which can be reverted by transfection with human dCK (Van der Wilt *et al*, 2000). The increased dCK activity is the most logical explanation for the collateral sensitivity in MDR cells, while the decreased dCDA activity may add to enhanced intracellular gemcitabine levels available for phosphorylation. However, the role of dCDA activity in gemcitabine sensitivity is still not clear (Bergman *et al*, 2002). The levels of both dCK mRNA and protein were also higher in 2R120 and 2R160 cells. Comparable differences in dCK levels have been associated previously with differences in gemcitabine sensitivity (Kroep *et al*, 2002). The increase in the activating enzyme was not only associated with increased retention of dFdCTP pools, but also with more gemcitabine incorporation into DNA.

Jensen *et al* (1997) previously described that daunorubicin- and VM-26-resistant MDR variants of human SCLC cell lines were also more sensitive to gemcitabine and to the structurally and functionally related deoxycytidine analogue ara-C. However, the extent of collateral sensitivity to gemcitabine and ara-C in the SCLC cell lines was less than that for the present cell lines, although in the SCLC line also an increased dCK activity was present (Bergman *et al*, 2001a). In addition to these cell lines, an increase in ara-C sensitivity was found in refractory AML cells displaying a P-gP or MRP overexpression after treatment with MDR drugs (Schuurhuis *et al*, 1995). It was suggested that exposure of cells to an MDR drug might select for a subpopulation of P-gP- or MRP-overexpressing cells with the more favourable enzymatic profile for cell survival. Our *in vitro* data suggest that the selection process may involve an increase in dCK.

In addition to the structural increase in dCK, Sasvári-Székely *et al* (1998) reported that in human lymphocytes, inhibition of DNA synthesis by 2-chloro-2′deoxyadenosine (Cl-Ado) resulted in a rapid, transient rise of dCK activity, which was however, not associated with an increase in the amount of dCK, while dCK mRNA levels even decreased. Apparently, dCK itself was activated, probably through a post-translation modification. Moreover, since dCK is a major enzyme in the supply of essential deoxynucleotides via the salvage pathway for DNA repair, its activation might be part of the cellular restoration process occurring after drug treatment. Unlike the transient increase in dCK activity in human lymphocytes during Cl-Ado exposure, the increase in dCK activity found in our NSCLC cell lines seemed to be stable. In 2R120 and SW1573 cells, 40% of the dCyd phosphorylation was the result of TK2 activity, while in 2R160 the TK2 activity was two-fold lower. Since TK2 only phosphorylates small 2′-substituted dCyd analogues and ara-C and gemcitabine are poor substrates (Wang *et al*, 1999), its role in the phosphorylation of gemcitabine might be limited, although its real contribution has not yet been established. A reduced TK2 activity might result in a higher dFdCTP/dCTP ratio in 2R160 cells than in SW1573 cells, which might contribute to an increased sensitivity to gemcitabine.

The diphosphate of gemcitabine (dFdCDP) inhibits ribonucleotide reductase (RNR), resulting in a decreased conversion of CDP to dCDP and eventually to a depletion of dCTP pools, which can favour dFdCTP incorporation into DNA (Baker *et al*, 1991; Plunkett *et al*, 1995). The dFdCTP accumulation and retention in several human tumour cell lines showed a relation with gemcitabine sensitivity *in vitro* (Ruiz van Haperen *et al*, 1994; Van Moorsel *et al*, 2000). dFdCTP is not only important as a DNA precursor, but also interferes with normal ribonucleotide metabolism, such as inhibition of CTP-synthetase and dCMP-deaminase leading to a depletion of CTP pools and indirectly a decrease of dCTP pools (Heinemann *et al*, 1990, 1995; Van Moorsel *et al*, 1999b, 2000). No difference was found in the retention of dFdCTP pools of SW1573 cells and its MDR variants after exposure to 1.0 *μ*M gemcitabine, which might be related to differences in nucleoside transport or 5′-nucleotidase (5NT) activity, known to oppose the action of deoxynucleoside kinases (Bergman *et al*, 2002). However, MDR variants retained dFdCTP pools longer than SW1573 cells after exposure to 0.1 *μ*M gemcitabine. After 24 h, no detectable dFdCTP pools were found in SW1573 cells, which might be related to the lower dCK activity or altered 5NT activity. The concentration-dependent [^3^H]gemcitabine incorporation into DNA and correlation of DNA damage with sensitivity is in agreement with previous studies in ovarian, colon and leukaemia cell lines (Huang *et al*, 1991; Ruiz van Haperen *et al*, 1993b). However, no relation was found between [^3^H]gemcitabine incorporation into RNA and sensitivity to gemcitabine, leaving a dubious role for RNA incorporation in gemcitabine toxicity (Bergman *et al*, 2002). Most likely, the increased dFdCTP retention, gemcitabine incorporation and increased sensitivity to gemcitabine are all downstream events of an increased dCK activity, underlining the pivotal role of dCK in gemcitabine sensitivity.

Verapamil is a strong inhibitor of P-gP, but only a partial inhibitor of MRP activity (Aszalos *et al*, 1999). A concentration of 10 *μ*M verapamil increased sensitivity to doxorubicin of MRP1-transfected NIH/3T3 mouse fibroblasts, but reversal was incomplete (Breuninger *et al*, 1995). Since we used a 2.5-fold higher verapamil concentration, MRP1 inhibition might be more effective. Reversal of collateral sensitivity to gemcitabine was found both in P-gP- and MRP1-overexpressing cells. We did not find a direct correlation between the extent of P-gP and MRP1 activity and sensitivity to gemcitabine or dCK activity. Apparently, a certain threshold level of P-gP or MRP1 activity is required for an increase in gemcitabine sensitivity, in contrast to the resistance to, for example, doxorubicin. The observation that verapamil reverted the collateral sensitivity of the MDR cells to gemcitabine suggests a possible relation between the presence of a certain membrane efflux pump activity, gemcitabine sensitivity and a transient regulation of dCK activity. One of these mechanisms may include regulation by protein kinase C (PKC) activity. Several studies report an increase in PKC activity in cells with an overexpression of P-gP or MRP (Beck *et al*, 1998; Ratnasinghe *et al*, 1998). P-glycoprotein and MRP are phosphorylated (Ma *et al*, 1995; Clavy *et al*, 1997); however, it is a matter of debate whether phosphorylation modulates the pump function (Ma *et al*, 1995; Smith and Zilfou, 1995; Clavy *et al*, 1997; Ratnasinghe *et al*, 1998). Since dCK may be phosphorylated by PKC, and exhibits a higher activity in the phosphorylated state (Wang and Kucera, 1994), PKC might play a role in the collateral sensitivity to gemcitabine of MDR cells.

Since P-gP and MRP act as an efflux pump for steroid hormones, such as cortisol, progesterone and aldosterone (Van Kalken *et al*, 1993; Mulder *et al*, 1996) and several studies reported a decrease in TK activity as a result of steroid hormone exposure in chicken embryo retina (Naray *et al*, 1977; Herzfield and Raper, 1980; Tesoriere *et al*, 1989), verapamil might inhibit the efflux of these compounds, leading to a transient downregulation of dCK. Clinically relevant concentrations of the steroid drug dexamethasone inhibited the effect of gemcitabine in cultured human glioma cells (Rieger *et al*, 1999). Moreover, dexamethasone decreased gemcitabine sensitivity of 2R120 and 2R160 cells, and decreased dCK activity in 2R160 cells, but only in the presence of verapamil (Bergman *et al*, 2001b).

In conclusion, MRP1- and P-gP-overexpressing cells were more sensitive to gemcitabine than their parental cells. This increased sensitivity was related to dCK and gemcitabine effects on DNA. Since relapsed tumours of patients after treatment with MDR drugs frequently display an MDR phenotype, screening for a P-gP or MRP1 overexpression, as a predictor of gemcitabine responsiveness, might be of clinical interest.

## References

[bib1] Abratt RP, Rezwoda W, Falkson G, Goedhals L, Hacking D (1994) Efficacy and safety profile of gemcitabine in non-small cell lung cancer. Phase II study. J Clin Oncol 12: 1535–1540 8040664 10.1200/JCO.1994.12.8.1535

[bib2] Aszalos A, Thompson K, Yin JJ, Ross DD (1999) Combinations of P-glycoprotein blockers, verapamil, PSC833, and cremophor act differently on the multidrug resistance associated protein (MRP) and on P-glycoprotein (P-gP). Anticancer Res 19: 1053–1064 10368654

[bib3] Baker CH, Banzon J, Bollinger JM, Stubbe J (1991) 2′-Deoxy-2′-methylenecytidine and 2′-deoxy-2′,2′-difluorodeoxycytidine 5′-diphosphates: potent mechanism-based inhibitors of ribonucleotide reductase. J Med Chem 34: 1879–1884 2061926 10.1021/jm00110a019

[bib4] Beck J, Bohnet B, Brugger D, Bader P, Dietl J, Scheper RJ, Kandolf R, Liu C, Niethammer D, Gekeler V (1998) Multiple gene expression analysis reveals distinct differences between G2 and G3 stage breast cancers, and correlations of PKC eta with mdr1, MRP and LRP gene expression. Br J Cancer 77: 87–91 9459150 10.1038/bjc.1998.13 PMC2151261

[bib7] Bergman AM, Giaccone G, Van Moorsel CJ, Mauritz R, Noordhuis P, Pinedo HM, Peters GJ (2000) Cross-resistance in the 2′,2′-difluorodeoxycytidine (gemcitabine) resistant human ovarian cancer cell line AG6000 to standard and investigational drugs. Eur J Cancer 36: 1974–1983 11000580 10.1016/s0959-8049(00)00246-x

[bib8] Bergman AM, Munch-Petersen B, Jensen PB, Sehested M, Veerman G, Voorn DA, Smid K, Pinedo HM, Peters GJ (2001a) Collateral sensitivity to gemcitabine (2′,2′-difluorodeoxycytidine) and cytosine arabinoside of daunorubicin- and VM-26-resistant variants of human small cell lung cancer cell lines. Biochem Pharmacol 61: 1401–1408 11331076 10.1016/s0006-2952(01)00627-x

[bib9] Bergman AM, Pinedo HM, Peters GJ (2001b) Steroids affect collateral sensitivity to gemcitabine of multidrug-resistant human lung cancer cells. Eur J Pharmacol 416: 19–24 11282108 10.1016/s0014-2999(01)00858-5

[bib10] Bergman AM, Pinedo HM, Peters GJ (2002) Determinants of resistance to 2′,2′-difluorodeoxycytidine (gemcitabine). Drug Resist Update 5: 19–33 10.1016/s1368-7646(02)00002-x 12127861

[bib6] Bergman AM, Pinedo HM, Jongsma APM, Brouwer M, Ruiz van Haperen VWT, Veerman G, Leyva A, Eriksson S, Peters GJ (1999) Decreased resistance to gemcitabine of cytosine arabinoside resistant myeloblastic murine and rat leukemia cell lines: role of altered activity and substrate specificity of deoxycytidine kinase. Biochem Pharmacol 57: 397–406 9933028 10.1016/s0006-2952(98)00318-9

[bib5] Bergman AM, Ruiz van Haperen VWT, Veerman G, Kuiper CM, Peters GJ (1996) Synergistic interaction between cisplatin and gemcitabine *in vitro*. Clin Cancer Res 2: 521–530 9816199

[bib11] Birnboim HC, Jevcak JJ (1981) Fluorometic method for rapid detection of DNA strand breaks in human white blood cells produced by low doses of radiation. Cancer Res 41: 1889–1892 7214357

[bib12] Borst P, Evers R, Kool M, Wijnholds J (2000) A family of drug transporters: the multidrug resistance-associated proteins. J Natl Cancer Inst 92: 1295–1302 10944550 10.1093/jnci/92.16.1295

[bib13] Braakhuis BJM, Ruiz van Haperen VWT, Boven E, Veerman G, Peters GJ (1995) Schedule dependent antitumor effect of gemcitabine in *in vivo* model systems. Semin Oncol 22(Suppl 11): 42–46 7481844

[bib14] Breuninger LM, Paul S, Gaughan K, Miki T, Chan A, Aaronson SA, Kruh GD (1995) Expression of multidrug resistance-associated protein in NIH/3T3 cells confers multidrug resistance associated with increased drug efflux and altered intracellular drug distribution. Cancer Res 55: 5342–5347 7585598

[bib15] Clavy JS, Horwitz SB, Orr GA (1997) Identification of the *in vivo* phosphorylation sites for acidic-directed kinases in murine mdr1b P-glycoprotein. J Biol Chem 272: 5909–5914 9038209 10.1074/jbc.272.9.5909

[bib16] Cornwell MM, Pastan I, Gottesman MM (1987) Certain calcium channel blockers bind specifically to multidrug resistant human KB carcinoma membrane vesicles and inhibit drug binding to P-glycoprotein. J Biol Chem 262: 2166–2170 2434476

[bib17] Davidson JD, Ma L, Iverson PW, Lesoon A, Jin S, Horwitz L, Gallery M, Slapak CA (2002) Human multi-drug resistance protein 5 (MRP5) confers resistance to gemcitabine. Proc Am Assoc Cancer Res 43: abstract 3868

[bib18] Endicott JA, Ling V (1989) The biochemistry of P-glycoprotein-mediated multidrug resistance. Ann Rev Biochem 58: 137–171 2570548 10.1146/annurev.bi.58.070189.001033

[bib19] Eriksson S, Kierszuk B, Munch-Petersen B, Oberg B, Johansson NG (1991) Comparison of the substrate specifities of human thymidine kinase 1 and 2 and deoxycytidine kinase toward antiviral and cytostatic nucleoside analogs. Biochem Biophys Res Commun 176: 586–592 2025274 10.1016/s0006-291x(05)80224-4

[bib20] Grant CE, Valdimarsson G, Hipfner DR, Almquist KC, Cole SPC, Deeley RG (1994) Overexpression of multidrug resistance-associated protein (MRP) increases resistance to natural product drugs. Cancer Res 54: 357–361 8275468

[bib21] Grant S, Turner A, Nelms P, Yanovich S (1995) Characterization of a multidrug resistant human erythroleukemia cell line (K562) exhibiting spontaneous resistance to 1-*β*-D-arabinofuranosylcytosine. Leukemia 9: 808–814 7769843

[bib22] Hatzis P, Al-Madhoon AS, Jüllig M, Petrakis TG, Eriksson S, Talianidis I (1998) The intracellular localization of deoxycytidine kinase. J Biol Chem 273: 30239–30243 9804782 10.1074/jbc.273.46.30239

[bib23] Heinemann V (2001) Gemcitabine: progress in the treatment of pancreatic cancer. Oncology 60: 8–18 11150902 10.1159/000055290

[bib24] Heinemann V, Hertel LW, Grindey GB, Plunkett W (1988) Comparison of the cellular pharmacokinetics and toxicity of 2′,2′-difluorodeoxycytidine and 1-*β*-D-arabinofuranosylcytosine. Cancer Res 48: 4024–4031 3383195

[bib25] Heinemann V, Schulz L, Issels RD, Plunkett W (1995) Gemcitabine: a modulator of intracellular nucleotide and deoxynucleotide metabolism. Semin Oncol 22(4 Suppl 11): 11–18 7481839

[bib26] Heinemann V, Xu Y-Z, Chubb S, Sen A, Hertel LW, Grindey GB, Plunkett W (1990) Inhibition of ribonucleotide reduction in CCRF-CEM cells by 2′,2′-difluorodeoxycytidine. Mol Pharmacol 38: 567–572 2233693

[bib27] Herzfield A, Raper SM (1980) Relative activities of thymidylate synthetase and thymidine kinase in rat tissues. Cancer Res 40: 744–750 7471094

[bib28] Huang P, Chubb S, Hertel LW, Grindey GB, Plunkett W (1991) Action of 2′,2′-difluorodeoxycytidine on DNA synthesis. Cancer Res 51: 6110–6117 1718594

[bib29] Jensen PB, Holm B, Sorensen M, Christensen IJ, Sehested M (1997) *In vitro* cross-resistance and collateral sensitivity in seven resistant small-cell lung cancer cell lines: preclinical identification of suitable drug partners to taxotere, taxol, topotecan and gemcitabine. Br J Cancer 75: 869–877 9062409 10.1038/bjc.1997.154 PMC2063407

[bib30] Keepers YP, Pizao PE, Peters GJ, Van Ark-Otte J, Winograd B, Pinedo HM (1991) Comparison of the sulforhodamine B protein and tetrazolium (MTT) assays for *in vitro* chemosensitivity testing. Eur J Cancer 27: 897–900 1834124 10.1016/0277-5379(91)90142-z

[bib31] Kuiper CM, Broxterman HJ, Baas F, Schuurhuis GJ, Haisma HJ, Scheffer GL, Lankelma J, Pinedo HM (1990) Drug transport variants without P-glycoprotein overexpression from a human squamous lung cancer cell line after selection with doxorubicin. J Cell Pharmacol 1: 35–41

[bib32] Kroep JR, Loves WJP, Van der Wilt CL, Alvarez E, Talianidis I, Boven E, Braakhuis BJM, Van Groeningen CJ, Pinedo HM, Peters GJ (2002) Pretreatment deoxycytidine kinase levels predict *in vivo* gemcitabine sensitivity. Mol Cancer Ther 1: 371–376 12477049

[bib33] Lincke CR, Van der Bliek AM, Schuurhuis GJ, Van der Velde-Koerts T, Smit JJM, Borst P (1990) Multidrug-resistance phenotype of human BRO melanoma cells transfected with a wild-type human mdr1 complementary DNA. Cancer Res 50: 1779–1785 1968359

[bib34] Louie KG, Hamilton TC, Winker MA, Behrens BC, Tsuruo T, Klecker RW, McKoy WM, Grotzinger KR, Myers CE, Young RC, Ozols RF (1986) Adriamycin accumulation and metabolism in adriamycin-sensitive and -resistant human ovarian cancer cell lines. Biochem Pharmacol 35: 467–472 3947382 10.1016/0006-2952(86)90221-2

[bib35] Lund B, Hansen OP, Theilade K, Hansen M, Neijt JP (1994) Phase II study of gemcitabine (2′,2′-difluorodeoxycytidine) in previously treated ovarian cancer patients. J Natl Cancer Inst 86: 1530–1533 7932808 10.1093/jnci/86.20.1530

[bib36] Ma L, Krishnamachary N, Center MS (1995) Phosphorylation of the multidrug resistance associated protein gene encoded protein P190. Biochemistry 34: 3338–3343 7880829 10.1021/bi00010a024

[bib37] Mackey JR, Mani RS, Selner M, Mowle D, Young JD, Belt JA, Crawford CR, Cass CE (1998) Functional nucleoside transporters are required for gemcitabine influx and manifestations of toxicity in cancer cell lines. Cancer Res 58: 4349–4357 9766663

[bib38] Mackey JR, Yao SY, Smith KM, Karpinski E, Baldwin SA, Cass CE, Young JD (1999) Gemcitabine transport in *Xenopus* oocytes expressing recombinant plasma membrane mammalian nucleoside transporters. J Natl Cancer Inst 91: 1876–1881 10547395 10.1093/jnci/91.21.1876

[bib39] Mulder HS, Pinedo HM, Timmer AT, Rao BR, Lankelma J (1996) Multidrug resistance-modifying components in human plasma with potential clinical significance. J Exp Therap Oncol 1: 13–22 9414384

[bib40] Naray A, Aranyi P, Foldes I, Horvath I (1977) Analysis of thymidine kinase actvity and glucocorticoid binding capacity in the thymuses of healthy and tumor bearing chickens. J Natl Cancer Inst 59: 1237–1241 903999 10.1093/jnci/59.4.1237

[bib41] Peters GJ, Wets M, Keepers YPAM, Oskam R, Van Ark-Otte J, Noordhuis P, Smid K, Pinedo HM (1993) Transformation of mouse fibroblasts with the oncogenes H-ras or trk is associated with pronounced changes in drug sensitivity and metabolism. Int J Cancer 54: 450–455 8509220 10.1002/ijc.2910540316

[bib42] Plunkett W, Huang O, Xu YZ, Heinemann V, Grunewald R, Ghandi V (1995) Gemcitabine: metabolism, mechanisms of action, and self-potentiation. Semin Oncol 22(Suppl 11): 42–46 7481842

[bib43] Ratnasinghe D, Phang JM, Yeh GC (1998) Differential expression and activity of phosphatases and protein kinases in adriamycin sensitive and resistant human breast cancer MCF-7 cells. Int J Oncol 13: 79–84 9625806 10.3892/ijo.13.1.79

[bib44] Rieger J, Durka S, Streffer J, Weller M (1999) Gemcitabine cytotoxicity of human malignant glioma cells: modulation by antioxidants, bcl-2 and dexamethasone. Eur J Pharmacol 365: 301–308 9988115 10.1016/s0014-2999(98)00883-8

[bib45] Ritzel MW, Ng AM, Yao SY, Graham K, Loewen SK, Smith KM, Ritzel RG, Mowles DA, Carpenter P, Chen XZ, Karpinski E, Hyde RJ, Baldwin SA, Cass CE, Young JD (2001) Molecular identification and characterization of novel human and mouse concentrative Na^+^-nucleoside cotransporter proteins (hCNT3 and mCNT3) broadly selective for purine and pyrimidine nucleosides (system cib). J Biol Chem 276: 2914–2927 11032837 10.1074/jbc.M007746200

[bib46] Rots MG, Willey JC, Jansen G, Van Zandwijk CH, Noordhuis P, DeMuth JP, Kuiper E, Veerman AJP, Pieters R, Peters GJ (2000) mRNA expression levels of methotrexate resistance related proteins in childhood leukemia as determined by a standardized competitive template based RT-PCR method. Leukemia 14: 2166–2175 11187907 10.1038/sj.leu.2401943

[bib47] Ruiz van Haperen VWT, Peters GJ (1994) New targets for pyrimidine antimetabolites for the treatment of solid tumours. II: Deoxycytidine kinase. Pharmacy World Sci 16: 104–112 10.1007/BF01880661 7980770

[bib48] Ruiz van Haperen VWT, Veerman G, Boven E, Noordhuis P, Vermorken JB, Peters GJ (1994) Schedule dependence of sensitivity of 2′,2′-difluorodeoxycytidine (gemcitabine) in relation to accumulation and retention of its triphosphate in solid tumor cell lines and solid tumors. Biochem Pharmacol 48: 1327–1339 7945430 10.1016/0006-2952(94)90554-1

[bib50] Ruiz van Haperen VWT, Veerman G, Braakhuis BJM, Vermorken JB, Boven E, Leyva A, Peters GJ (1993a) Deoxycytidine kinase and deoxycytidine deaminase activities in human tumour xenografts. Eur J Cancer 29A: 2132–2137 8297652 10.1016/0959-8049(93)90048-k

[bib49] Ruiz van Haperen VWT, Veerman G, Vermorken JB, Peters GJ (1993b) 2′,2′-Difluoro-deoxycytidine (gemcitabine) incorporation into RNA and DNA from tumour cell lines. Biochem Pharmacol 46: 762–766 8363650 10.1016/0006-2952(93)90566-f

[bib51] Sasvári-Székely M, Spasokoukotskaja T, Szóke M, Csapó Z, Turi Á, Szántó I, Eriksson S, Staub M (1998) Activation of deoxycytidine kinase during inhibition of DNA synthesis by 2-chloro-2′-deoxyadenosine (Cladribine) in human lymphocytes. Biochem Pharmacol 56: 1175–1179 9802328 10.1016/s0006-2952(98)00108-7

[bib52] Schuurhuis GJ, Broxterman HJ, Ossenkoppele GJ, Baak JPA, Eekman CA, Kuiper CM, Feller N, Van Heijningen THM, Klumper E, Pieters R, Lankelma J, Pinedo HM (1995) Functional multidrug resistance phenotype associated with combined overexpression of PgP/MDR1 and MRP together with 1-*β*-D-arabinofuranosylcytosine sensitivity may predict clinical response in acute myeloid leukemia. Clin Cancer Res 1: 81–93 9815890

[bib53] Shen DW, Fojo A, Chin JE, Roninson IB, Richert N, Pastan I, Gottesman MM (1986) Human multidrug resistant cell lines: increased mdr1 expression can precede gene amplification. Science 232: 643–645 3457471 10.1126/science.3457471

[bib54] Skehan P, Storeng R, Scudiero D, Monks A, McMahon J, Vistica D, Warren JT, Bokesch H, Kenney S, Boyd MR (1990) New calorimetric cytotoxicity assay for anticancer drug screening. J Natl Cancer Inst 82: 1107–1112 2359136 10.1093/jnci/82.13.1107

[bib55] Smith CD, Zilfou JT (1995) Circumvention of P-glycoprotein mediated multiple drug resistance by phosphorylation modulators is independent of protein kinases. J Biol Chem 270: 28145–28152 7499304 10.1074/jbc.270.47.28145

[bib56] Tesoriere G, Vento R, Taibi G, Calvaruso G, Schiavo MR (1989) Biochemical aspects of chick embryo retina development: the effects of glucocorticosteroids. J Neurochem 52: 1487–1494 2709012 10.1111/j.1471-4159.1989.tb09198.x

[bib57] Van der Wilt CL, Kroep JR, Bergman AM, Loves WJP, Alvarez E, Talianidis I, Van Groeningen CJ, Pinedo HM, Peters GJ (2000) The role of deoxycytidine kinase in gemcitabine cytotoxicity. Adv Exp Med Biol 486: 287–290 11783501 10.1007/0-306-46843-3_56

[bib58] Van der Wilt CL, Visser GW, Braakhuis BJ, Wedzinga R, Noordhuis P, Smid K, Peters GJ (1993) *In vitro* antitumour activity of *cis*- and *trans*-5-fluoro-5,6-dihydro-6-alkoxy-uracils; effects on thymidylate synthesis. Br J Cancer 68: 702–707 8398696 10.1038/bjc.1993.413 PMC1968601

[bib59] Van Kalken CK, Broxterman HJ, Pinedo HM, Feller N, Dekker H, Lankelma J, Giaccone G (1993) Cortisol is transported by the multidrug resistance gene product P-glycoprotein. Br J Cancer 67: 284–289 8094292 10.1038/bjc.1993.54 PMC1968171

[bib60] Van Moorsel CJA, Pinedo HM, Veerman G, Bergman AM, Kuiper CM, Vermorken JB, Van der Vijgh WJ, Peters GJ (1999a) Mechanisms of synergism between cisplatin and gemcitabine in ovarian and non-small-cell lung cancer cell lines. Br J Cancer 80: 981–990 10362105 10.1038/sj.bjc.6690452 PMC2363050

[bib61] Van Moorsel CJA, Pinedo HM, Veerman G, Guechev A, Smid K, Loves WJP, Veerman JB, Postmus PE, Peters GJ (1999b) Combination chemotherapy studies with gemcitabine and etoposide in non-small cell lung and ovarian cancer cell lines. Biochem Pharmacol 57: 407–415 9933029 10.1016/s0006-2952(98)00316-5

[bib62] Van Moorsel CJA, Bergman AM, Veerman G, Voorn DA, Ruiz van Haperen VWT, Kroep JR, Pinedo HM, Peters GJ (2000) Differential effects of gemcitabine on ribonucleotide pools of twenty-one solid tumour and leukaemia cell lines. Biochim Biophys Acta 1474: 5–12 10699484 10.1016/s0304-4165(99)00209-3

[bib63] Wang LM, Kucera GL (1994) Deoxycytidine kinase is phosphorylated *in vitro* by protein kinase C alpha. Biochem Biophys Acta 1224: 161–167 7981228 10.1016/0167-4889(94)90186-4

[bib64] Wang L, Munch-Petersen B, Herrstrom Sjoberg A, Hellman U, Bergman T, Jornvall H, Eriksson S (1999) Human thymidine kinase 2: molecular cloning and characterisation of the enzyme activity with antiviral and cytostatic nucleoside substrates. FEBS Lett 443: 170–174 9989599 10.1016/s0014-5793(98)01711-6

[bib65] Zaman GJR, Flens MJ, Van Leusden MR, De Haas M, Mulder HS, Lankelma J, Scheper RJ, Baas F, Broxterman HJ, Borst P (1994) The human multidrug resistance-associated protein MRP is a plasma membrane drug-efflux pump. Proc Natl Acad Sci USA 91: 8822–8826 7916458 10.1073/pnas.91.19.8822 PMC44698

[bib66] Zaman GJR, Versantvoort CHM, Smit JJM, Eijdens EWHM, De Haas M, Smith AJ, Broxterman HJ, Mulder NH, De Vries EGE, Baas F, Borst P (1993) Analysis of the expression of MRP, the gene for a new putative transmembrane drug transporter, in human multidrug resistant lung cancer cell lines. Cancer Res 53: 1747–1750 8467491

